# Cytomegalovirus Colitis in a Patient With Ulcerative Colitis on Vedolizumab Monotherapy

**DOI:** 10.7759/cureus.35473

**Published:** 2023-02-25

**Authors:** Yaser Meeralam, Bushra Al Qurashi, Assel Al Masoudi, Talal L Alhejaili, Mohammed Khayat, Anas M Aljoaid, Wallaa Al Harthi, Waleed A Hafiz, Mohammed K Shariff

**Affiliations:** 1 Digestive and Liver Health Center, King Abdullah Medical City, Makkah, SAU; 2 Internal Medicine Department, King Abdullah Medical City, Makkah, SAU; 3 Department of Medicine, College of Medicine, Umm Al-Qura University, Makkah, SAU

**Keywords:** vedolizumab, inflammatory bowel disease, extra-intestinal manifestation, ulcerative colitis, cmv

## Abstract

Cytomegalovirus (CMV) is a human herpes-type virus with variable clinical manifestations. Infections in immunocompetent patients are usually asymptomatic or mild, and severe infections are generally seen in immunosuppressed individuals. CMV colitis is not uncommon in patients with ulcerative colitis (UC) and is mostly associated with the use of steroids, immunomodulators like azathioprine, and biologics like infliximab, which have systemic immunosuppressive effects. Vedolizumab is an anti-integrin antibody that is gut-selective without any systemic effects. We report an unusual presentation of a female patient with UC who had concomitant CMV colitis and erythema nodosum, who was on vedolizumab, and not on any steroids or other immunosuppressants. She responded well to anti-viral treatment and steroids.

## Introduction

Cytomegalovirus (CMV) infection is infrequently seen in patients with ulcerative colitis (UC), and it is most commonly associated with the use of immunosuppressive drugs such as corticosteroids in inflammatory bowel disease (IBD) [1,2]. CMV colitis in UC is difficult to differentiate from the underlying disease due to overlapping symptoms and requires high clinical suspicion along with histopathology to confirm the diagnosis. In the untreated, the prognosis is very poor and response to anti-viral therapy determines the outcome [1].

Vedolizumab is a biologic effective in treating UC; it has a good safety profile as its action is limited to the gut with negligible systemic effects. The use of vedolizumab was not associated with the risk of CMV colitis based on accumulated data from clinical trials and retrospective studies. CMV colitis with vedolizumab was mainly seen in those taking other concomitant immunosuppressants including steroids.

In this report, we present an interesting case of CMV colitis in a UC patient taking vedolizumab as a monotherapy, which was successfully treated with anti-viral therapy and steroids.

## Case presentation

A 44-year-old woman with UC diagnosed six years prior presented with a one-week history of bloody diarrhea with a frequency of 8-10 times per day and associated generalized abdominal pain. She also noted the formation of new-onset, painful, and red skin rash over her lower extremities. She was maintained on vedolizumab 300 mg intravenous (IV) infusion every eight weeks. Her physical examination was unremarkable except for tender erythematous nodules over her lower extremities.

Her initial laboratory investigations revealed microcytic hypochromic anemia [hemoglobin of 8.7 g/dL (normal range: 12-15 g/dL) and mean corpuscular volume of 77 fL (normal range: 80-100 fL)] and high inflammatory markers [C-reactive protein of 4.2 mg/dL (normal level: <0.5 mg/dL)]. Though the skin biopsy of the rash was nonspecific, the dermatological assessment of the skin rash was consistent with erythema nodosum. The stool for ova/parasite, culture, and clostridium difficile toxins was negative.

**Figure 1:**
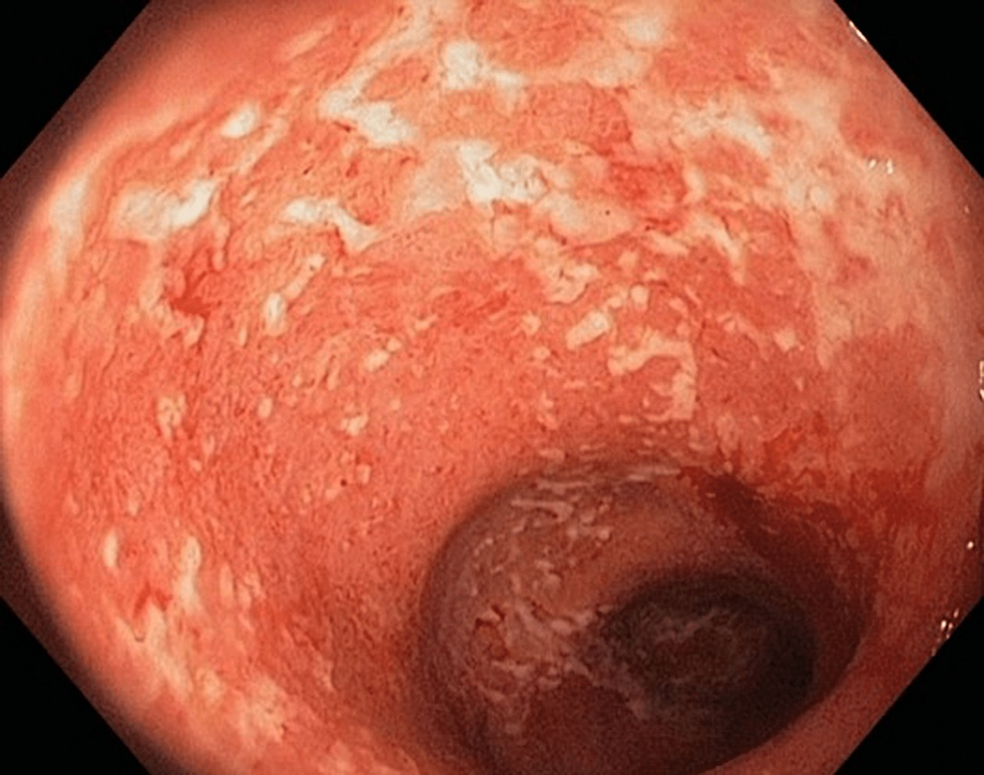
Severe colonic inflammation with whitish exudate

Colonoscopy was performed and showed contentious erythematous mucosa with loss of vascularity and whitish exudate extending from the rectum up to the splenic flexure suggestive of severe left-sided colitis (Figure 1).

**Figure 2:**
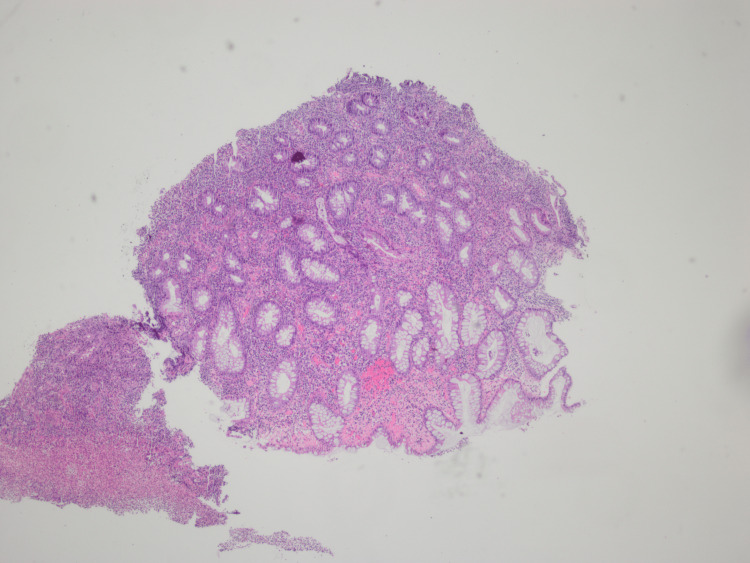
Ulcerated colonic tissue with marked chronic active colitis, cryptitis, and regenerative epithelium

Histopathologic examination of the biopsied tissues demonstrated moderate left-sided chronic active colitis with ulcer slough and crypt disarray with no evidence of inclusion bodies (Figure 2).

Tissue biopsy for CMV quantitative polymerase chain reaction (PCR) was positive with a viral load of 66,750 IU/ml and CMV serology showed positive Immunoglobulin G (IgG) and negative immunoglobulin M (IgM).

During her hospitalization, the patient was started on IV methylprednisolone 30 mg IV every 12 hours, and her skin lesions resolved in four days. However, her bloody diarrhea persisted. Hence, she was started on an induction dose of ganciclovir 5 mg/kg IV every 12 hours for CMV colitis and her IV methylprednisolone was switched to oral prednisolone with tapering of the dose by 5 mg every 12 weeks. The patient reported remarkable improvement in her bloody diarrhea within 10 days of ganciclovir initiation. After about three weeks of hospitalization, her symptoms completely resolved and she was discharged home on a maintenance dose of valganciclovir 900 mg per oral every 12 hours for 21 days.

Three weeks after her discharge, she was re-assessed in the clinic where she reported complete resolution of her bloody diarrhea.

## Discussion

CMV is a Herpesviridae DNA virus that causes primary or secondary infection, the latter due to the reactivation of the dormant virus. It is usually asymptomatic in immunocompetent patients. However, reactivation of CMV in immunocompromised hosts can result in pneumonia, retinitis, and colitis, the latter being the most common presentation [3]. 

The prevalence of CMV infection among patients with UC ranges from 2 to 38% [4]. UC is strongly associated with CMV colitis with the severity of the disease and steroid refractoriness significantly increasing the risk of CMV colitis [5]. Other factors increasing the risk of CMV colitis include the use of aminosalicylic acid, pancolitis, female gender, older age, azathioprine, and cyclosporine [5]. The role of infliximab has been contentious, with some suggesting no association with CMV colitis in UC while others demonstrating a significant association [5,6]. International societies do recommend the exclusion of CMV in patients with moderate to severe UC, especially in patients with steroid-refractory diseases [7].

The symptoms of diarrhea, rectal bleeding, and abdominal pain are encountered in both CMV colitis and underlying active UC. In addition, though the characteristic colonoscopic features of CMV colitis are punched-out longitudinal ulcers, they could vary from erythema, with the erosions to ulcers making it difficult to distinguish from active UC, thereby making the diagnosis of CMV colitis challenging. A high clinical suspicion is important in patients at risk. There are several methods to diagnose CMV infections, including serum antibody measurement, antigenemia assay, CMV PCR, histological hematoxylin and eosin, immunohistochemical staining, and tissue CMV PCR. A positive CMV IgG antibody indicates previous exposure to CMV, while the presence of CMV IgM confirms acute infection or reactivation of CMV. Serum antibodies against CMV antigens are not associated with CMV colitis, and CMV antigenemia assay and serum PCR detect CMV colitis with low sensitivity. The presence of inclusion bodies in histological hematoxylin and eosin and immunohistochemical staining is diagnostic for CMV colitis, and the most highly sensitive and specific test for CMV colitis is tissue CMV PCR [8].

In the present case, the young woman presented a UC flare while on a maintenance dose of vedolizumab as monotherapy. Hence, IV methylprednisolone was initiated while awaiting histopathological results with no clinical response. CMV colitis was diagnosed by colonic tissue PCR, a gold-standard diagnostic tool. Anti-viral therapy was then started, and all her symptoms resolved following this.

Vedolizumab is an anti-integrin biologic that inhibits leucocyte trafficking in the gut without any systemic effect [9]. This tissue-specific action makes vedolizumab a much safer option and a preferred drug over other biologics for the treatment of UC [9]. Accumulated data from multiple large studies did demonstrate vedolizumab to be a safe drug and reported only two to four cases of CMV colitis in UC. Though the full details about these cases were not given, the risk of CMV colitis was not increased and was more likely to be associated with those UC patients taking steroids and other immunosuppressants along with vedolizumab [10,11]. This indicated that well-known factors other than vedolizumab were driving the CMV infection. Going a step further, one study did suggest that vedolizumab was in fact effective in treating CMV colitis in UC [12]. Our patient, despite being on vedolizumab monotherapy without any other systemic immunosuppressant therapy, developed CMV colitis. The possible explanation for this CMV reactivation could either be the active underlying UC itself or vedolizumab exerting a local and/or systemic immunosuppressant’s action.

## Conclusions

CMV infection should be considered in all complicated UC patients or patients with persistent colitis not responding to immunosuppressive medication. Though vedolizumab is considered a safe biologic, its potential for systemic effect should be further explored and should be considered as a possible risk factor for CMV colitis in UC.
